# Eradicated and bygone Hansen’s disease with unusual features (leprosy)

**DOI:** 10.11604/pamj.2022.42.245.35598

**Published:** 2022-07-29

**Authors:** Chaitanya Ajay Kulkarni, Prasad Pramod Dhage

**Affiliations:** 1 Department of Community Health Physiotherapy, Ravi Nair Physiotherapy College, Datta Meghe Institute of Medical Sciences, Sawangi, Wardha, Maharashtra, India

**Keywords:** Hansen’s disease, leprosy, *Mycobacterium leprae*

## Image in medicine

A 70-years pueblo female was admitted to the dermatology and venerology department with serious complaints of pain, swelling, yellowish discharge from fingers, fever, malaise, and blackish discoloration of the skin of the left lower limb, and arthralgia for 1 month. As per the history of the relative, she was hypertensive and diabetic for 15 years and on the medications for the same. There is no associated family history. There is a significant history of loss of appetite and weight loss. On further physical examinations, the patient was febrile at 38.8°C (102 F), with multiple sites of skin infections with loss of sensation in the upper limbs as well as lower limbs with multiple ulcerated lesions at bilateral hands and fingers with yellowish discharge (A) and lower limb. According to the histopathology and hematology report, the patient had an active infection with *Mycobacterium leprae* and was diagnosed with a case of Hansen´s disease (leprosy). As the infection was spreading progressively to the left foot and ankle patient was operated for left below-knee amputation (B). The patient was then referred to the physical therapy department for further therapeutic management.

**Figure 1:**
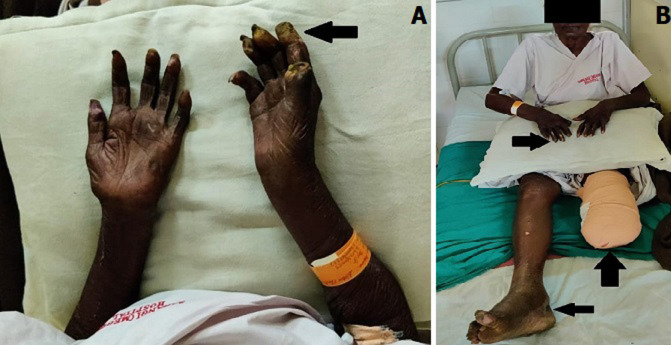
erythematous skin lesion with yellowish discharge from fingers (A); multiple skin lesions on fingers, foot, and left below-knee amputated leg (B)

